# Supplementary material for: Promoting physical activity in glioma patients: Insights from Dutch healthcare professionals
Source: Neurooncol Pract. 2026 Feb 27;13(4):685–93. doi: 10.1093/nop/npag020 (PMC13365158; doi:10.1093/nop/npag020)
Supplement: npag020_Supplementary_Data [file npag020_supplementary_data.zip › Supplementary figure captions.docx]

# Supplementary figure captions

**Supplementary figure 1.** (**A**) Perceived barriers among healthcare professionals who rarely or sometimes provide proactive physical activity advice (N=13). (**B**) Perceived need for additional training or support in the same group.

**Supplementary figure 2.** Perceived need for additional training or support to promote physical activity among healthcare professionals with more than 10 years of experience (N = 26; **A**) and 10 years or less (N = 29; **B**). Perceived barriers to promoting physical activity among healthcare professionals with more than 10 years of experience (N = 26; **C**) and 10 years or less (N = 29; **D**).

**Supplementary figure 3.** (**A**) Perceived barriers among healthcare professionals who indicated no need for specific guidelines for glioma patients. (**B**) Perceived need for additional training or support in the same group.
