# Supplementary material for: Promoting physical activity in glioma patients: Insights from Dutch healthcare professionals
Source: Neurooncol Pract. 2026 Feb 27;13(4):685–93. doi: 10.1093/nop/npag020 (PMC13365158; doi:10.1093/nop/npag020)
Supplement: npag020_Supplementary_Data [file npag020_supplementary_data.zip › Supplementary health care survey_clean.docx]

**Supplementary Table 1. English translation of the original Dutch survey**

| **Healthcare professional profile** | | |
| --- | --- | --- |
| *Question* | | *Answer (open or closed)* |
| 1 | What is your gender? | - Male - Female - Non-binary - Other, namely |
| 2 | What is your age? |  |
| 3 | What is your specialty? | - Neurologist - Neurosurgeon - Oncologist - Radiotherapist - Nurse - Nurse specialist - (Neuro)psychologist - Other, namely: |
| 4 | How many years have you been working in this specialty? |  |
| 5 | In which center do you work? |  |

| **Current care of glioma patients** | | |
| --- | --- | --- |
| *Question* | | *Answer (open or closed)* |
| 6 | Do you ever receive questions from patients regarding physical activity/exercise/sports/fitness? | Never – rarely – sometimes – often – very often |
| 7 | What kind of questions do you receive? |  |
| 8 | At which stage of the disease do you mainly receive these questions? | *Multiple answers possible*   - Before treatment - During treatment - After treatment - Other, namely: |
| 9 | Do you ever advise patients on your own initiative to be more physically active? | Never – rarely – sometimes – often – very often |
| 10 | If so, what kind of advice do you give? |  |
| 11 | To what extent do you experience barriers in giving physical activity advice/promoting physical activity? | No barriers at all – few barriers – neutral – some barriers – many barriers |
| 12 | What barriers do you experience? | *Multiple answers possible*   - Not feeling competent - Limited knowledge - Workload - Not part of routine - Time - Availability of informative material - Not convinced by the literature - Not convinced of the relevance - Not a priority - Limited referral options - Other, namely: |
| 13 | What is needed to remove these barriers? In other words, what support or resources would you need to be able to give physical activity advice? |  |
| 14 | Would you like additional training or support to promote physical activity in glioma patients? For example, education or supporting materials. | Strongly agree – agree – neutral – disagree – strongly disagree |
| 15 | In your opinion, how and when should physical activity best be integrated into the care plan for glioma patients? |  |

| **Physical activity in glioma patients** | | |
| --- | --- | --- |
| *Question* | | *Answer (open or closed)* |
| 16 | What do you think are the potential benefits of physical activity for patients with glioma? |  |
| 17 | And what are the potential disadvantages? |  |
| 18 | Do you think physical activity can help reduce symptoms (such as fatigue, pain, anxiety, etc.)? | Strongly agree – agree – neutral – disagree – strongly disagree |
| 19 | Do you think physical activity can help improve quality of life? | Strongly agree – agree – neutral – disagree – strongly disagree |
| 20 | To what extent do you consider physical activity safe for glioma patients? Can you elaborate on your answer? |  |
| 21 | Do you think there is sufficient evidence for the effectiveness of physical activity for glioma patients? | Strongly agree – agree – neutral – disagree – strongly disagree |
| 22 | Would you recommend physical activity as part of the care for glioma patients? | Strongly agree – agree – neutral – disagree – strongly disagree |

| **Physical activity guidelines** | | |
| --- | --- | --- |
| *Question* | | *Answer (open or closed)* |
| 23 | Are you familiar with the general physical activity guidelines (for the general population)? | - Yes - No |
| 24 | Would you like to have a physical activity guideline specifically for glioma patients? | - Yes - No |
| 25 | What do you do yourself in terms of physical activity in a week? In what form and how often? |  |

**Supplementary Table 2. Thematic overview of open-ended questions and key answers**

| **Open-ended questions** | | **Theme** | **Type of answers** | |
| --- | --- | --- | --- | --- |
| **Patient questions** | | | | |
| *What type of questions do you receive?* | | Permission | Is it allowed to exercise/be physically active?; Is it possible for individuals with reduced physical capacity, low energy levels, or epilepsy? | |
|  |  | How to | How to stay or become physically fit?; What type of exercise, at what intensity, and at which point in time? Are there any recommendations? | |
|  |  | Advantages | Is it wise or beneficial, for example, in reducing side effects or fatigue, improving tolerance to treatment, even influencing tumor growth, or improving mental health? | |
| **Recommendations** | | | | |
| *What type of recommendations do you give?* | | Frequency, type, intensity | Stay active every day; walking or other low intensity activities; do what you can, something is better than nothing; make it your routine. | |
|  |  | Relevance | PA is important for both physical and mental health, improves quality of life; helps cope with treatment side effects, and supports recovery. | |
|  |  | Personalization | Tailor advice to individual situations and abilities; adjust activity based on symptoms, energy levels; seek guidance or supervision (physiotherapy) if needed. | |
| **Barriers and support** | | | | |
| *What would it take to remove barriers? In other words, what support or resources would you need to be able to promote PA?* | | Information and education | Brochures, informative materials, guidelines, and websites; evidence; education; advice should be tailored to the patient’s abilities. | |
|  |  | Access and referral | Need for a clear referral network, regional map of physiotherapists, or easier pathways to supervised exercise programs. | |
|  |  | Financial help | Insurance coverage for physiotherapy or supervised exercise | |
| **(Dis)advantages** | | | | |
| *In your opinion, what are the potential advantages of PA for patients with glioma?* | | Functioning and fitness | Improving physical condition and muscle strength; preserving independence and mobility. | |
|  |  | Mental health | Improved mood, less anxiety and depression; increased self-confidence and sense of control; distraction, relaxation, and daily structure. | |
|  |  | Quality of life | Overall well-being; more energy, less fatigue; better daily routine, and social interaction; focus on abilities. | |
|  |  | Treatment benefits | Better tolerance and completion of treatments; fewer side effects; possible benefit for survival and recurrence risk (although evidence is limited). | |
| *What are the potential disadvantages?* | | Overexertion and fatigue | Risk of overexertion, with poor balance between activity and rest; not enough energy left for other activities; overexertion during intensive treatment. | |
|  |  | Psychological pressure | Feelings of guilt if unable to be active; sense of “having to” be active; frustration or disappointment when something is not possible. | |
|  |  | Safety | Increased risk of falls, especially with paresis, epilepsy, or unsafe activities; risk of injury; unstable epilepsy as a barrier. | |
|  |  | Practical barriers | Time investment, extra burden during limited life expectancy; costs for professional guidance; not accessible for everyone due to limited energy, support network, or resources; lack of motivation. | |
| **Safety** | |  |  | |
| *To what extent do you think PA is safe for glioma patients?* | | Adaptation | PA is generally considered safe, especially when tailored to individual abilities, symptoms and treatment phase; should be adjusted for neurological deficits, epilepsy, or other limitations. | |
|  |  | Supervision | Safety is enhanced with professional supervision for patients at higher risk; patients and families should be informed about limits. | |
|  |  | Type and intensity | Low- to moderate-intensity activity are usually safe, while high-impact or intensive sports may require extra caution. | |
| **Clinical care** | | | | |
| *How and when might PA best be integrated into the care plan for glioma patients in your opinion?* | When | | | From the onset of the disease; throughout the care trajectory; need to be adapted during the trajectory; after treatment (mentioned once); at specific moments such as specialist consultations. |
|  | How | | | Structured advice for at home; discussed regularly and included in written information and patient consultations; advice should be tailored; support from professionals should be offered when needed. |

*PA = physical activity*

**Supplementary Table 3. Post hoc analysis comparing nurses and neurologists**

| **Questions** | **Group (N)** | **Answers (%)** | | | | |
| --- | --- | --- | --- | --- | --- | --- |
|  |  | **Very often** | **Often** | **Sometimes** | **Rarely** | **Never** |
| Do you receive questions about PA? | 1 (18) | 22.2 | 50.0 | 27.8 | 0 | 0 |
|  | 2 (19) | 5.3 | 47.4 | 42.1 | 5.3 | 0 |
| Do you give PA recommendations? | 1 (18) | 16.7 | 72.2 | 11.1 | 0 | 0 |
|  | 2 (19) | 10.5 | 57.9 | 26.3 | 5.3 | 0 |
|  |  | **No barriers at all** | **Few barriers** | **Neutral** | **Some barriers** | **Many barriers** |
| Do you experience barriers in promoting PA? | 1 (18) | 61.1 | 27.8 | 11.1 | 0 | 0 |
|  | 2 (19) | 36.8 | 36.8 | 26.3 | 0 | 0 |
|  |  | **Totally agree** | **Agree** | **Neutral** | **Disagree** | **Totally disagree** |
| Do you need support to be able to promote PA? | 1 (18) | 0 | 44.4 | 44.4 | 5.6 | 5.6 |
|  | 2 (19) | 21.1 | 36.8 | 26.3 | 15.8 | 0 |

*Group 1 = nurses; Group 2 = neurologists; PA = physical activity*
