# Supplementary figures and images for: Promoting physical activity in glioma patients: Insights from Dutch healthcare professionals
Source: Neurooncol Pract. 2026 Feb 27;13(4):685–93. doi: 10.1093/nop/npag020 (PMC13365158; doi:10.1093/nop/npag020)

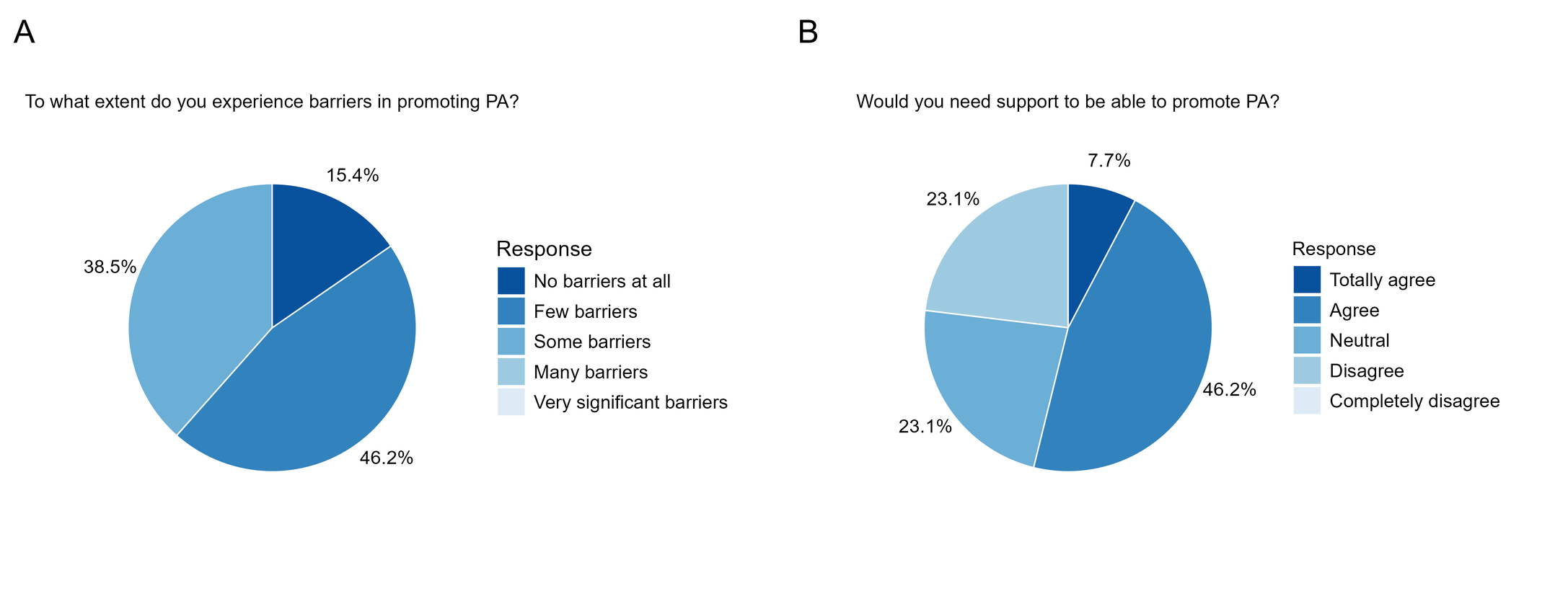

Supplement: npag020_Supplementary_Data [file npag020_supplementary_data.zip › Supplementary figure 1.tiff]

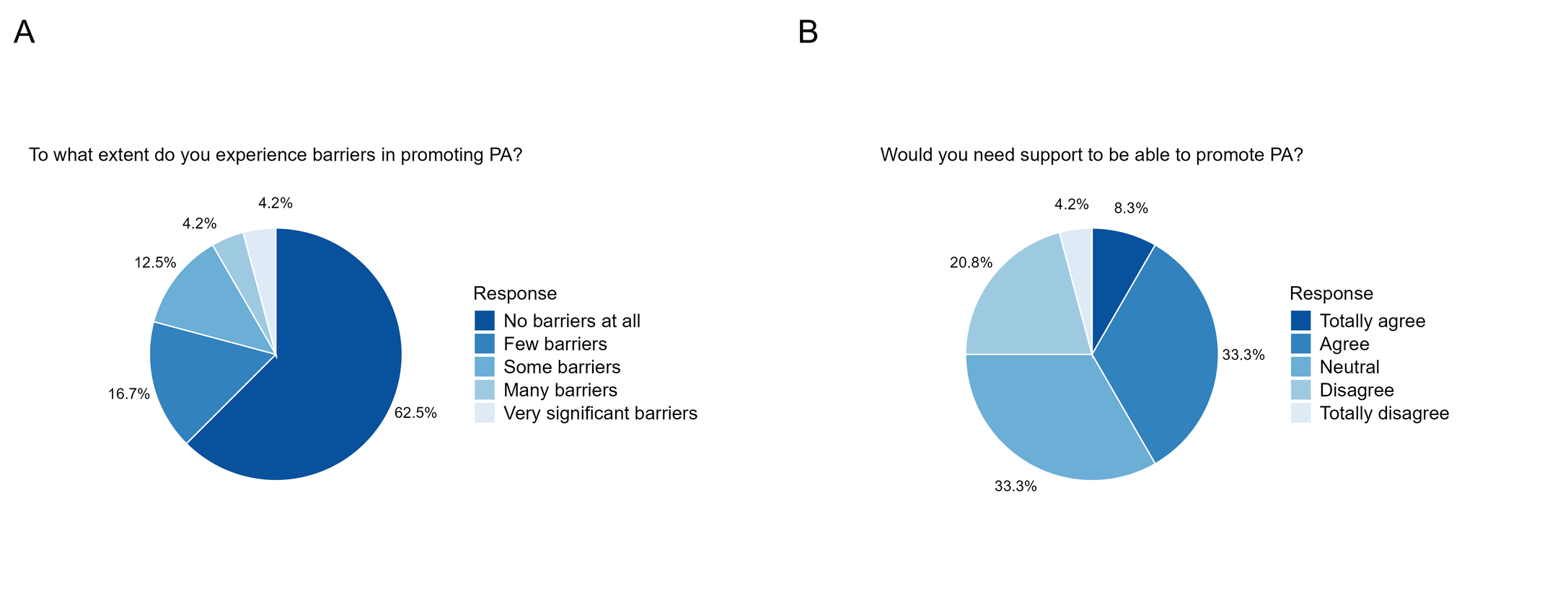

Supplement: npag020_Supplementary_Data [file npag020_supplementary_data.zip › Supplementary figure 3.tiff]
